# Quantitative determination by real-time PCR of four vaginal *Lactobacillus species*, *Gardnerella vaginalis *and *Atopobium vaginae *indicates an inverse relationship between *L. gasseri *and *L. iners*

**DOI:** 10.1186/1471-2180-7-115

**Published:** 2007-12-19

**Authors:** Ellen De Backer, Rita Verhelst, Hans Verstraelen, Mohammed A Alqumber, Jeremy P Burton, John R Tagg, Marleen Temmerman, Mario Vaneechoutte

**Affiliations:** 1 Department of Clinical Chemistry, Microbiology & Immunology, Ghent University Hospital, UGent, Ghent, Belgium; 2 Department of Obstetrics & Gynaecology, Ghent University Hospital, UGent, Ghent, Belgium; 3 Department of Microbiology and Immunology, University of Otago, Dunedin, New Zealand; 4 BLIS Technologies Ltd., Centre for Innovation, University of Otago, Dunedin, New Zealand

## Abstract

**Background:**

Most studies of the vaginal microflora have been based on culture or on qualitative molecular techniques. Here we applied existing real-time PCR formats for *Lactobacillus crispatus*, *L. gasseri *and *Gardnerella vaginalis *and developed new formats for *Atopobium vaginae*, *L. iners *and *L. jensenii *to obtain a quantitative non culture-based determination of these species in 71 vaginal samples from 32 pregnant and 28 non-pregnant women aged between 18 and 45 years.

**Results:**

The 71 vaginal microflora samples of these women were categorized, using the Ison and Hay criteria, as refined by Verhelst *et al*. (2005), as follows: grade Ia: 8 samples, grade Iab: 10, grade Ib: 13, grade I-like: 10, grade II: 11, grade III: 12 and grade IV: 7.

*L. crispatus *was found in all but 5 samples and was the most frequent *Lactobacillus *species detected. A significantly lower concentration of *L. crispatus *was found in grades II (p < 0.0001) and III (p = 0.002) compared to grade I. *L. jensenii *was found in all grades but showed higher concentration in grade Iab than in grade Ia (p = 0.024). *A. vaginae *and *G. vaginalis *were present in high concentrations in grade III, with log_10 _median concentrations (log_10 _MC), respectively of 9.0 and 9.2 cells/ml. Twenty (38.5%) of the 52 *G. vaginalis *positive samples were also positive for *A. vaginae*. In grade II we found almost no *L. iners *(log_10 _MC: 0/ml) but a high concentration of *L. gasseri (*log_10 _MC: 8.7/ml). By contrast, in grade III we found a high concentration of *L. iners (*log_10 _MC: 8.3/ml) and a low concentration of *L. gasseri (*log_10 _MC: 0/ml). These results show a negative association between *L. gasseri *and *L. iners *(r = -0.397, p = 0.001) and between *L. gasseri *and *A. vaginae *(r = -0.408, p < 0.0001).

**Conclusion:**

In our study we found a clear negative association between *L. iners *and *L. gasseri *and between *A. vaginae *and *L. gasseri*. Our results do not provide support for the generally held proposition that grade II is an intermediate stage between grades I and III, because *L. gasseri*, abundant in grade II is not predominant in grade III, whereas *L. iners*, abundant in grade III is present only in low numbers in grade II samples.

## Background

Bacterial vaginosis (BV) is considered to be the most frequent vaginal infectious disorder in women of childbearing age. Prevalences of 4.9% to 36% have been reported from European and American studies [1]. The condition is symptomatic in half of the women and also represents a psychological burden. More importantly, this disturbed vaginal microflora can cause serious sequelae such as non-gonococcal, non-chlamydial PID (Pelvic Inflammatory Disease) [2,3], postpartum endometritis [4] and preterm birth [5-7]. Indeed, 40% of the cases of spontaneous preterm labor and preterm birth are thought to be associated with BV [8]. BV has also been associated with increased susceptibility to HIV and to genital tract infection with *Chlamydia trachomatis *and *Neisseria gonorrhoeae *[9-11].

BV is characterized by an overgrowth of many different, mostly anaerobic, bacteria. *Gardnerella vaginalis *has been considered to be the most characteristic microorganism in BV, but its pathogenic role was unclear, until recently, when the establishment of bacterial biofilm as a potential cause of recurrent BV [12], with a predominant presence of *G. vaginalis*, has clearly established a pathogenic role for this bacterium. Also recently, *Atopobium vaginae *has been strongly associated with BV, independently by different groups [13-15] and with biofilm in BV [12]. The suggestion that its association with BV is stronger than that of *G. vaginalis*, basically because *G. vaginalis *is more frequently detected in a normal vaginal microflora than *A. vaginae *[14] was confirmed by more recent studies [16,17]. The insight that biofilm is formed in BV [12] has strong explanatory power with regard to the high recurrence rate of BV, despite initial relief after antibiotic treatment [18] and is in perfect correlation with the presence of the characteristic cells or 'clue cells', which are vaginal epithelial cells covered with layers of bacteria.

Four species of lactobacilli are now considered to be predominantly linked to the vaginal microflora: *Lactobacillus crispatus*, *L. jensenii*, *L. gasseri *and *L. iners*, with the latter only recently being recognized as it was long overlooked because it does not grow on De Man Rogosa Sharpe agar, the medium typically used to culture lactobacilli [19,20].

In 1983 Spiegel *et al*. [21] devised a method for the diagnosis of bacterial vaginosis by direct Gram stain. Nugent *et al*. [22] proposed a modification of these criteria. By counting the different cell types (*Lactobacillus *spp., *Gardnerella vaginalis*/*Bacteroides *spp., *Mobiluncus *spp.) a score between zero and ten is obtained, whereby a score of 7 or higher corresponds to bacterial vaginosis and a score between 3 and 7 is considered intermediate between undisturbed vaginal microflora and bacterial vaginosis. Ison and Hay [23] suggested a simplification of this time-consuming approach, through an estimation of the ratios of the observed cellular types rather than the determination of the exact number of the bacteria and distinguished five grades (0 (no bacteria), I (normal), II (intermediate), III (bacterial vaginosis) and IV (streptococci)). Verhelst *et al*. [24] recently suggested, on the basis of Gram stain, terminal restriction fragment length polymorphism analysis of the 16S rRNA gene (T-RFLP) and molecular identification (tDNA-PCR) of cultured isolates, a new classification of the undisturbed vaginal microflora (grade I) whereby grade I microflora could be split up into four categories, designated grade Ia, Ib, Iab and I-like. Grade Ia was shown to contain predominantly *L. crispatus*, grade Ib predominantly *L. gasseri *and *L. iners*, grade Iab a mixture of these three species and grade I-like *Bifidobacterium *spp. rather than *Lactobacillus *spp. Grade I-like microflora, of which the Gram stain at first glance indicates the presence of lactobacilli, was considered as not representative for normal vaginal micoflora, but as probably an unrecognized type of disturbed vaginal microflora [25] that had previously not been distinguished from a healthy vaginal microflora.

In this study, we developed real-time PCR primers for *L. iners*, *L. jensenii *and *A. vaginae *and used these, together with described real-time PCR formats for *L. crispatus *[26]*, L. gasseri *[26] and *G. vaginalis *[27], in an attempt to quantify some of the important bacterial species in the normal and disturbed vaginal microflora.

## Results

### Grading of vaginal microflora

In this study, Gram-stained smears of 71 vaginal swabs from 60 women, 32 of whom were pregnant, were examined microscopically. For 11 pregnant women the Gram stain based grading of their vaginal microflora differed between two trimesters and therefore swabs from both trimesters were included. Eight women had a vaginal microflora categorized by Gram stain as grade Ia, 10 as grade Iab, 13 as grade Ib, 10 as grade I-like, 11 as grade II, 12 as grade III and 7 as grade IV, according to criteria described previously [24].

### Primer development and real-time PCR format

**Figure 1:**
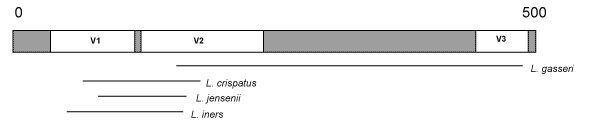
Localisation of the primers used in this study on the 16S rRNA gene.

Primers for real-time PCR for *Atopobium vaginae*, *Lactobacillus iners *and *L. jensenii *were developed during this study (Tables 1, 2, 3). Primer positions relative to the 16S rRNA gene of *Escherichia coli *are presented in Figure 1. Table 4 lists all primers used and the real-time PCR formats. No cross-reactivity was detected with DNA from any of the tested species, including closely-related *Lactobacillus *and *Atopobium *species. In addition we found no cross reactivity for *Bifidobacterium bifidum*, *B. breve *and *B. infantis *with the *G. vaginalis *primers developed by Zariffard *et al*. [27].

**Table 1:** Alignment of the 16S rRNA gene primers of *L. iners *with the sequences of other important vaginal lactobacilli

**Forward primer (position 70–85)**	5'																			3'	
*L. iners*		**G**	**T**	**C**	**T**	**G**	**C**	**C**	**T**	**T**	**G**	**A**	**A**	**G**	**A**	**T**	**C**	**G**	**G**		
*L. crispatus*		**A**	**C**	**.**	**.**	**.**	**.**	**.**	**C**	**C**	**A**	**T**	**.**	**.**	**T**	**C**	**T**	**.**	**.**		
*L. gasseri*		**A**	**C**	**.**	**.**	**.**	**.**	**.**	**C**	**A**	**A**	**G**	**.**	**.**		**C**	**T**	**.**	**.**		
*L. jensenii*		**A**	**C**	**.**	**.**	**.**	**.**	**.**	**C**	**.**	**T**	**.**	**.**	**.**	**T**	**C**	**T**	**.**	**.**		
*L. vaginalis*		**A**	**C**	**.**	**.**	**.**	**.**	**.**	**C**	**.**	**.**	**.**	**.**	**.**	**C**	**G**	**G**	**.**	**.**		
																					
**Reverse primer (position 228–210)**	5'																				3'
*L. iners*		**A**	**C**	**A**	**G**	**T**	**T**	**G**	**A**	**T**	**A**	**G**	**G**	**C**	**A**	**T**	**C**	**A**	**T**	**C**	
*L. crispatus*		**.**	**.**	**.**	**C**	**.**	**.**	**A**	**G**	**C**	**.**	**C**	**T**	**.**	**.**	**.**	**.**	**G**	**.**	**T**	
*L. gasseri*		**.**	**.**	**.**	**C**	**.**	**.**	**A**	**G**	**C**	**.**	**C**	**T**	**.**	**.**	**.**	**.**	**G**	**.**	**T**	
*L. jensenii*		**.**	**.**	**.**	**C**	**.**	**.**	**A**	**G**	**C**	**.**	**C**	**T**	**.**	**.**	**.**	**.**	**G**	**.**	**T**	
*L. vaginalis*		**.**	**.**	**.**	**C**	**C**	**.**	**A**	**G**	**C**	**.**	**C**	**T**	**.**	**.**	**.**	**.**	**G**	**.**	**T**	

**Table 2:** Alignment of the 16S rRNA gene primers of *L. jensenii*, with the sequences of other important vaginal lactobacilli

**Forward primer (position 117–137) **	5'																						3'
*L. jensenii*		**C**	**C**	**T**	**T**	**A**	**A**	**G**	**T**	**C**	**T**	**G**	**G**	**G**	**A**	**T**	**A**	**C**	**C**	**A**	**T**	**T**	
*L. crispatus*		**.**	**.**	**C**	**A**	**T**	**.**	**.**	**.**	**.**	**.**	**.**	**.**	**.**	**.**	**.**	**.**	**.**	**.**	**.**	**C**	**.**	
*L. gasseri*		**.**	**.**	**A**	**A**	**G**	**.**	**.**	**A**	**.**	**.**	**.**	**.**	**.**	**.**	**.**	**.**	**A**	**.**	**.**	**C**	**C**	
*L. iners*		**.**	**.**	**A**	**A**	**G**	**.**	**.**	**A**	**T**	**C**	**.**	**.**	**.**	**.**	**.**	**.**	**A**	**.**	**.**	**C**	**C**	
*L. vaginalis*		**.**	**.**	**.**	**G**	**.**	**.**	**.**	**C**	**G**	**G**	**.**	**.**	**.**	**.**	**.**	**.**	**A**	**.**	**.**	**.**	**C**	
																							
**Reverse primer (position 207–187) **	5'																						3'
*L. jensenii*		**A**	**C**	**G**	**C**	**C**	**G**	**C**	**C**	**T**	**T**	**T**	**T**	**A**	**A**	**A**	**C**	**T**	**T**	**C**	**T**	**T**	
*L. crispatus*		**.**	**.**	**.**	**.**	**.**	**.**	**.**	**.**	**.**	**.**	**.**	**.**	**.**	**.**	**.**	**A**	**G**	**C**	**T**	**G**	**A**	
*L. gasseri*		**.**	**-**	**A**	**.**	**.**	**A**	**T**	**.**	**.**	**.**	**.**	**.**	**.**	**.**	**.**	**.**	**.**	**C**	**T**	**A**	**G**	
*L. iners*		**.**	**-**	**A**	**.**	**.**	**A**	**T**	**.**	**.**	**.**	**.**	**.**	**.**	**.**	**.**	**.**	**A**	**G**	**T**	**.**	**G**	
*L. vaginalis*		**.**	**A**	**A**	**.**	**.**	**A**	**T**	**.**	**.**	**.**	**.**	**G**	**.**	**.**	**.**	**.**	**G**	**A**	**A**	**A**	**A**	

**Table 3:** Alignment of the 16S rRNA gene primers of *A. vaginae *with the sequences of the described *Atopobium *species

**Forward primer (position 1006–1025)**	5'																					3'
*A. vaginae*		**G**	**G**	**T**	**G**	**A**	**A**	**G**	**C**	**A**	**G**	**T**	**G**	**G**	**A**	**A**	**A**	**C**	**A**	**C**	**T**	
*A. fossor*		**.**	**.**	**.**	**.**	**.**	**.**	**.**	**.**	**G**	**.**	**C**	**.**	**.**	**.**	**.**	**.**	**.**	**G**	**T**	**C**	
*A. minutum*		**.**	**.**	**.**	**.**	**.**	**.**	**.**	**.**	**G**	**.**	**C**	**.**	**.**	**.**	**.**	**.**	**.**	**G**	**T**	**C**	
*A. parvulum*		**.**	**.**	**.**	**.**	**.**	**.**	**.**	**.**	**G**	**.**	**C**	**.**	**.**	**.**	**.**	**.**	**.**	**G**	**T**	**C**	
*A. rimae*		**.**	**.**	**.**	**.**	**.**	**.**	**.**	**.**	**G**	**.**	**C**	**.**	**.**	**.**	**.**	**.**	**.**	**G**	**T**	**C**	
																						
**Reverse primer (position 1282–1265)**	5'																				3'	
*A. vaginae*		**A**	**T**	**T**	**C**	**G**	**C**	**T**	**T**	**C**	**T**	**G**	**C**	**T**	**C**	**G**	**C**	**G**	**C**	**A**		
*A. fossor*		**.**	**.**	**.**	**G**	**.**	**.**	**.**	**.**	**A**	**C**	**T**	**T**	**.**	**.**	**.**	**.**	**A**	**A**	**G**		
*A. minutum*		**.**	**.**	**.**	**G**	**.**	**.**	**.**	**.**	**A**	**C**	**T**	**T**	**.**	**.**	**.**	**.**	**A**	**A**	**G**		
*A. parvulum*		**.**	**.**	**.**	**.**	**.**	**.**	**.**	**C**	**A**	**C**	**C**	**.**	**.**	**.**	**.**	**.**	**.**	**G**	**G**		
*A. rimae*		**.**	**.**	**.**	**.**	**.**	**.**	**.**	**C**	**G**	**C**	**C**	**.**	**.**	**T**	**.**	**.**	**.**	**G**	**G**		

**Table 4:** Overview of the primer sequences and PCR conditions as used in this study

**Specificity**	**Name**	**Primer sequence (5'-3')**	**16S rDNA position^a ^(5'-3')**	**Cycling conditions**	**Reference**
*A. vaginae*	ATOVAGRT3Fw	GGTGAAGCAGTGGAAACACT	1006–1025	10' 95°C, (15" 95°C, 20" 62°C, 40" 72°C) × 40	This study
	ATOVAGRT3Rev	ATTCGCTTCTGCTCGCGCA	1282–1265		
*G. vaginalis*	F-GV1	TTACTGGTGTATCACTGTAAGG	16S-23S spacer	10' 95°C, (45" 94°C, 45" 55°C, 45" 72°C) × 50	[27]
	R-GV3	CCGTCACAGGCTGAACAGT	16S-23S spacer		
*L. crispatus*	LcrisF	AGCGAGCGGAACTAACAGATTTAC	65–89	10' 95°C, (15" 95°C, 1' 60°C) × 40	[26]
	LcrisR	AGCTGATCATGCGATCTGCTT	205–185		
*L. gasseri*	LactoF	TGGAAACAGRTGCTAATACCG	157–177	10' 95°C, (15" 95°C, 1' 60°C) × 40	[26]
	LgassR	CAGTTACTACCTCTATCTTTCTTCACTAC	470–442		
*L. iners*	InersFw	GTCTGCCTTGAAGATCGG	70–85	10' 95°C, (1' 95°C, 1' 55°C, 1' 65°C) × 35	This study
	InersRev	ACAGTTGATAGGCATCATC	228–210		
*L. jensenii*	LABJENTR2Fw	CCTTAAGTCTGGGATACCATT	117–137	10' 95°C, (15" 95°C, 10" 54°C, 30" 72°C) × 40	This study
	LABJENRT2Rev	ACGCCGCCTTTTAAACTTCTT	207–187		

^a^Relative to the position in the *Escherichia coli *16S rDNA sequence.

Using Qiagen extracted DNA from pure cultures; the detection limit for *A. vaginae *is 1.3 pg/ml, for *G. vaginalis *43.2 pg/ml, for *L. crispatus *4.7 pg/ml, for *L. gasseri *10.7 pg/ml, for *L. iners *6.4 pg/ml and for *L. jensenii *15.0 pg/ml.

### Qualitative results

Table 5 presents the overall presence of the six species studied in the different grades of vaginal microflora. *L. crispatus *was found in all but 5 samples (93%) and was the most frequent *Lactobacillus *species detected. *L. jensenii *was found in 33 samples (46%) but especially in grade Iab where it was present in 8 of 10 samples (80%).

**Table 5:** Percentage of samples of each grade for which the tested species were detected

	**Grade Ia**	**Grade Iab**	**Grade Ib**	**Grade I**	**Grade I-like**	**Grade II**	**Grade III**	**Grade IV**	**Total (%)**
Number of samples	8	10	13	31	10	11	12	7	100
*A. vaginae*	38	20	15	23	30	18	83	14	32
*G. vaginalis*	88	50	61.5	65	70	63	100	29	68
*L. crispatus*	100	100	100	100	80	82	92	100	93
*L. gasseri*	88	90	85	87	60	100	33	43	72
*L. iners*	50	90	92	81	70	27	100	86	75
*L. jensenii*	13	80	46	48	60	55	50	0	47

Forty-eight samples (68%) were positive for *G. vaginalis *and 20/48 (42%) of these were also positive for *A. vaginae*. Only three samples were positive for *A. vaginae *but negative for *G. vaginalis*. All grade III samples were positive for *G. vaginalis *and 10/13 (83%) were also positive for *A. vaginae*.

*L. gasseri *was detected in 52/71 samples (73%) and 27/31 (87%) were grade I, 7/10 (70%) grade I-like, 11/11 (100%) grade II, 4/12 (33%) grade III and 3/7 (43%) grade IV.

*L. iners *was detected in 53/71 samples (75%) from which 25/31 (81%) were grade I, 7/10 (70%) grade I-like, 3/11 (27%) grade II, 12/12 (100%) grade III and 6/7 (86%) grade IV.

### Quantitative results

**Figure 2:**
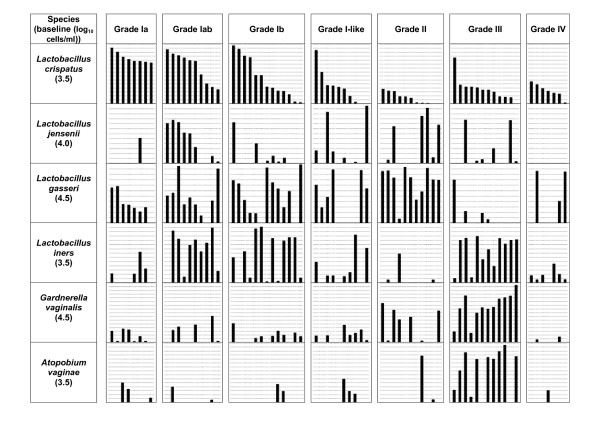
**Overview of the results obtained with real-time PCR for *L. crispatus*, *L. jensenii*, *L. gasseri*, *L. iners*, *G. vaginalis *and *A. vaginae***. x-axis: each bar represents a different sample. y-axis: logarithmic scale of cells/ml. Spaces in between horizontal lines represent log_10 _0.5 differences. Detection limits are 3.5 for *L. crispatus*, 4.0 for *L. jensenii*, 4.5 for *L. gasseri*, 3.5 for *L. iners*, 4.5 for *G. vaginalis *and 3.5 for *A. vaginae*. Data sorted per grade according to decreasing concentration of *L. crispatus*.

Figure 2 and Table 6 give an overview of the results obtained with real-time PCR for four vaginal *Lactobacillus *species and for *Gardnerella vaginalis *and *Atopobium vaginae*.

**Table 6:** Quantitative determination of the six species in the different grades expressed as Median log_10 _cells/ml (Interquartile Range)

	**Grade I**	**Grade I-like**	**Grade II**	**Grade III**	**Grade IV**
*A. vaginae*	0 (0 – 0)	0 (0 – 4.7)	0 (0 – 0)	9.0 (4.4 – 9.7)	0 (0 – 0)
*G. vaginalis*	5.1 (0 – 6.0)	5.4 (0 – 5.9)	4.7 (0 – 8.8)	9.2 (8.8 – 10.7)	0 (0 – 4.9)
*L. crispatus*	8.8 (5.6 – 9.5)	5.5 (2.8 – 6.2)	4.2 (3.6 – 5.1)	5.2 (4.3 – 5.6)	5.1 (4.7 – 5.9)
*L. gasseri*	6.6 (5.6 – 7.9)	6.4 (0 – 8.3)	8.7 (7.4 – 9.3)	0 (0 – 5.3)	0 (0 – 9.2)
*L. iners*	6.5 (3.7 – 8.6)	4.3 (3.9 – 4.5)	0 (0 – 3.8)	8.3 (5.6 – 8.7)	4.3 (3.9 – 4.5)
*L. jensenii*	4.0 (0 – 6.0)	4.3 (0 – 6.3)	4.3 (0 – 7.9)	2.1 (0 – 5.2)	0 (0 – 0)

The median log_10 _cells/ml were expressed as per 1 ml elution buffer.

We found a significantly higher level of *L. crispatus *in grade I (log_10 _Median Concentration (MC) = 8.8 cells/ml) compared to the other grades, i.e. II: 4.2, p < 0.0001, III: 5.2, p = 0.002, IV: 5.1, p = 0.004 and I-like: 5.5, p = 0.014. *L. crispatus *was also present in significantly higher amounts in grade III compared to grade II (p = 0.023).

*L. crispatus *was present in high levels (i.e. at least log_10 _8.5 cells/ml) in all 8 grade Ia samples (log_10 _MC = 9.0 cells/ml), in most (i.e. 6/10) grade Iab samples (log_10 _MC = 8.9 cells/ml), but only in 3/12 grade Ib samples (log_10 _MC = 5.6 cells/ml).

*L. jensenii *was found in all grades but showed higher concentration in grade Iab (log_10 _MC = 7.0 cells/ml) than in grade Ia (log_10 _MC = 0 cells/ml, p = 0.024)

The level of *L. gasseri *in grade I (log_10 _MC = 6.6 cells/ml) was significantly lower than in grade II (log_10 _MC = 8.7 cells/ml, p < 0.01) and significantly higher than in grade III (log_10 _MC = 0 cells/ml, p < 0.0001). The quantity of *L. gasseri *in grade II was significantly higher than in grade III (p < 0.0001) and than in grade IV (log_10 _MC = 0 cells/ml, p < 0.05).

The level for *L. iners *did not differ either between grades I and III or between grades I and I-like. *L. iners *was present in grade II (log_10 _MC = 0 cells/ml) in significantly lower amounts than in grade III (log_10 _MC = 8.3 cells/ml, p < 0.0001), in grade I (log_10 _MC = 6.5 cells/ml, p < 0.0001), in grade IV (log_10 _MC = 4.3 cells/ml, p < 0.05) and in grade I-like (log_10 _MC = 4.3 cells/ml, p < 0.05).

The concentrations of *A. vaginae *and *G. vaginalis *were significantly higher in grade III (respectively log_10 _MC = 9.0 cells/ml and 9.2 cells/ml) than in grade I (respectively log_10 _MC = 0 cells/ml and 5.1 cells/ml, p < 0.0001), grade II (respectively log_10 _MC = 0 cells/ml and 4.7 cells/ml p < 0.005), grade IV (respectively log_10 _MC = 0 cells/ml and 0 cells/ml, p < 0.005 and p < 0.0001) and grade I-like (respectively log_10 _MC = 0 cells/ml and 5.4 cells/ml, p < 0.005 and p < 0.0001). Concentrations of *G. vaginalis *were also higher in grade I than in grade IV (p < 0.05).

Significant positive correlations were found in our data set between *L. jensenii *and *L. gasseri *(*r *= 0.244, p < 0.05), between *G. vaginalis *and *L. iners *(*r *= 0.351, p < 0.05) and between *G. vaginalis *and *A. vaginae *(*r *= 0.469, p < 0.0001). Negative correlations were found between *L. gasseri *and *L. iners *(*r *= -0.397, p = 0.001) and between *A. vaginae *and *L. gasseri (r *= -0.408, p < 0.0001).

### Gram staining of *L. iners *and *L. gasseri*

**Figure 3:**
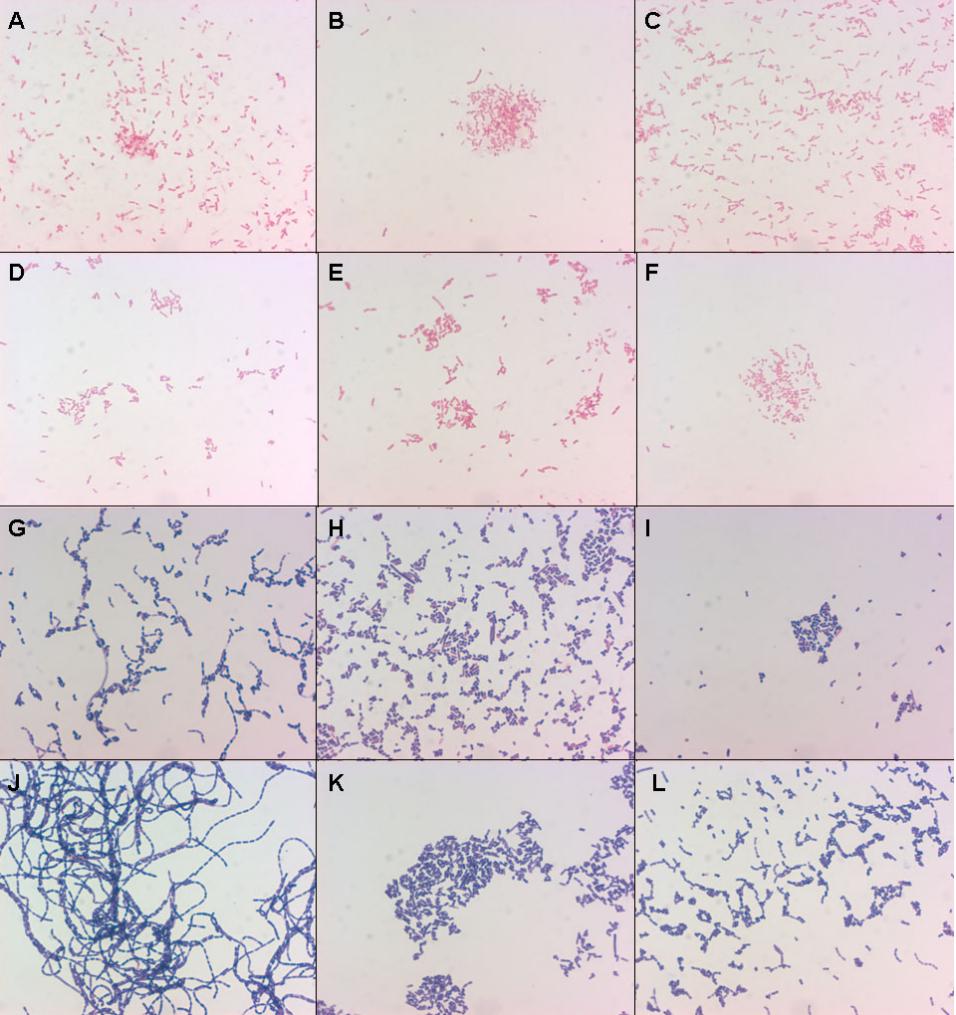
**Microscopic images (100 ×) of Gram stains of *L. iners *and *L. gasseri***. A-F: *L. iners*; A: BVS11, B: FB04-04, C: FB06-01, D: FB17-07, E: FB94-05b and F: FB30-01AN; G-L: *L. gasseri*: FB02-02, H: FB33-01, I: FB34-01AN, J: FB89-3, K: FB103-2, L: PB88-T2-1.

Figure 3 represents the Gram stains of 6 strains of *L. gasseri *and 6 strains of *L. iners*. Gram stains of *L. iners *and *L. gasseri *showed that many of these strains displayed pleiomorphic cell morphology, i.e. different cell types could be observed within one microscopic field. Also, the cell morphologies of strains differed within the same species. Most strikingly, all *L. iners *strains had a relatively Gram-negative stain appearance and were mostly very short rods. By contrast, the *L. gasseri *were clearly Gram-positive in all cases. Some strains of *L. gasseri *formed streptobacillar chains, consisting of long to extra-long rods.

### MIC of *L. gasseri *and *L. iners *for clindamycin

The clindamycin MIC value for ten strains of *L. iners *and *L. gasseri *was determined using the agar dilution method. Eight of the ten *L. iners *had a MIC value of 0.125 μg/ml, one strain had a MIC value of 2 μg/ml and one strain of >8 μg/ml. For *L. gasseri *one strain had a MIC value of 0.5 μg/ml, two strains had MIC values of 2 μg/ml, six strains had MIC values of 4 μg/ml and for one strain the MIC was more than 8 μg/ml. In summary, 80% of *L. iners *isolates were inhibited by 0.125 μg of clindamycin/ml, with MIC_50 _= 0.125 μg/ml, whereas 90% of the *L. gasseri *isolates was resistant to 1 μg of clindamycin/ml, with MIC_50 _= 4 μg/ml.

### Mutual inhibition of *L. gasseri *and *L. iners*

No inhibition zones were observed with the techniques we used.

## Discussion

In this study we used real-time PCR to obtain a quantitative estimation of the presence of the four predominant *Lactobacillus *species known to occupy the vaginal econiche and of *Atopobium vaginae *and *Gardnerella vaginalis*.

Real-time PCR has been applied previously for the description of changes in the vaginal microflora. Zariffard *et al*. [27] and Sha *et al*. [28,29] used real-time PCR on frozen cervicovaginal lavage samples to quantify the presence of *Mycoplasma hominis*, *G. vaginalis *and the combined presence of *L. crispatus *and *L. jensenii*, in comparison with the microscopic interpretation of the Gram stain according to the criteria of Nugent [22]. Bradshaw *et al*. [16] applied real-time PCR for *A. vaginae *for a follow-up study of recurrent BV before and after treatment with oral metronidazole. Ferris *et al*. [17] applied real-time PCR for *A. vaginae *to samples of six BV patients before and after treatment with a topical metronidazole gel. Thus far, only one group (Byun *et al*. [26]), studying advanced dental caries, used real-time PCR to quantify the presence of specific *Lactobacillus *species.

Compared to microscopy and culture, molecular methods such as PCR may provide less observer-dependent and culture medium dependent information and a more direct and detailed view of the quantity of certain species in a sample. E.g., *A. vaginae *was only recently recognized as strongly linked to disturbed vaginal microflora because of its fastidious growth requirements *in vitro*. A possible additional advantage of PCR, compared to culture, is that it permits detection of dead or metabolically inactive bacteria, and as such it can be applied for the detection of bacteria in a dormant state, as is the case in biofilms, which occur also in BV as has been shown by means of FISH [12].

Besides culture-associated bias, difficulties with regard to the standardization of vaginal sampling make quantification of the vaginal microflora cumbersome and make bacterial counts reported in different publications difficult to compare. Some groups have attempted to standardize sampling by cervicovaginal lavage (CVL), whereby the volume of saline that is recovered from the vagina can be measured and used for calibration of the bacterial load/ml. Drawbacks are that CVL samples also from the cervix and does not represent a pure vaginal sample. Also it cannot be excluded that biofilm, that may be present in cases of BV, is recalcitrant with regard to dissolving in the saline that is used and is not sampled in the same manner as loosely associated microflora. In the condition of BV there may be more vaginal cell debris so it is feasible that there is more non-bacterial material present in the sample. In conclusion, vaginal sampling remains difficult to standardize. In this study we used unweighted vaginal swabs taken by the same gynecologist so there is no standardization for the volume of the sample, but standardization of the way samples are taken.

Besides sampling bias and culture bias also real-time PCR may be biased, because the conversion of the PCR positivity threshold value to a concentration of bacteria, depends on the manner the standard curve is calibrated. The standard curve, which is prepared by extracting DNA from a dense bacterial suspension and by serial tenfold dilution of this DNA extract, can be compared to i) the bacterial colony count, as determined by plating out tenfold serial dilutions of the initial suspension, or to ii) the number of bacterial genomes in the initial suspension, determined on the basis of measurement of the DNA-concentration present in the extract from the initial suspension. We found differences between calculation of number of genomes by measurement of DNA-concentration and determination of number of cells by dilution culture from less to one log_10 _unit (*L. jensenii*) to more than 3 log_10 _units (*L. crispatus*) (data not presented). A possible explanation may be that species like *L. jensenii *are more easily cultured on blood plates than for example *L. crispatus *and therefore show a lower discrepancy between DNA-concentration and culture. In conclusion, when preparation of the standard curve in this study had been based on cultured cells, *L. jensenii *would have been overestimated relative to the other species.

### *L. crispatus *and *L. jensenii*

*L. crispatus *can be found in samples of all grades but the log_10 _median concentration/ml (MC) of this species is much higher in grade I (8.8) compared to grades II (4.2) and III (5.2). Although the difference between the log_10 _quantity of *L. crispatus *in grade Ia and grade Ib is not significantly different (p = 0.096), possibly because of the low sample size, a different profile is observed for these grades (Figure 2 and 3).

*L. crispatus *is present in high inoculum (i.e. at least) in all 8 grade Ia samples (log_10 _MC 9.0), in most (i.e. 6/10) grade Iab samples (log_10 _MC 8.9), but only in 3/12 grade Ib samples (log_10 _MC = 5.6). Our study confirms that a grade Ia microflora is characterized by high concentrations of *L. crispatus*, whereas grades Iab and Ib consist of a more diverse microflora, with different *Lactobacillus *species present in comparable amounts.

Also, in grade Ib, high concentrations of *L. crispatus *can be found but on Gram stain especially long and small *Lactobacillus *cell types, which were suggested to be more typical for *L. gasseri *[24], are observed.

Based on cloning of the 16S rDNA library from the indigenous microbiota of three women with normal vaginal microflora, Verhelst *et al*. [14] found an almost pure population of *L. jensenii *in one of them and concluded that this species was associated with a normal vaginal microflora, but in this study (71 swabs) we can not correlate *L. jensenii *with either normal or disturbed vaginal microflora since it is present sporadically in samples of all grades, and in both high and low numbers. Its presence in 8/10 grade Iab samples, compared to 1/8 grade Ia samples is striking (p = 0.024).

### *G. vaginalis *and *A. vaginae*

A positive correlation between *G. vaginalis *and *A. vaginae *could be established (p < 0.0001). *G. vaginalis *can be found in all grades but in much higher concentration in a disturbed vaginal microflora. Delaney *et al*. [30] performed a comparative study in which they quantified *G. vaginalis*, *Lactobacillus *spp., *Prevotella *spp. and *Peptostreptococcus *spp. in vaginal swabs by means of culture. The mean log_10 _number of *G. vaginalis *in BV was determined to be 9.64 cfu/g, which may compare well to the value we obtained, i.e. 9.2 cells/ml in grade III.

*A. vaginae *is found less frequently than *G. vaginalis *in non-grade III samples, but in high concentrations in 10 of the 12 grade III samples, confirming that the presence of *A. vaginae *seems to be a diagnostically more valuable marker for BV than the presence of *G. vaginalis*, as suggested by Verhelst *et al*. [14] and confirmed by several other studies [31-33]. A recent study using T-RFLP for the characterization of normal (n = 20) and BV (n = 50) microflora found that the terminal restriction fragment (TRF) with the length to that corresponding of *A. vaginae *was present in the vagina of 48 of the 50 women with BV (grade II and III lumped together) and absent from all 20 women with normal microflora [32]. Bradshaw *et al*. [16] and Ferris *et al*. [17] monitored the changes in the concentration of *A. vaginae *before and after treatment with metronidazole and both found that very high vaginal concentrations of *A. vaginae *were predictive for patients for whom treatment failed partially or completely, although Bradshaw *et al*. [16] found a significant reduction of *A. vaginae *after metronidazole treatment. This might be due to a lower susceptibility of some strains of *A. vaginae *for metronidazole [34] but a more plausible explanation might be the existence of biofilm as shown by Swidsinski *et al*. [12], and in which *G. vaginalis *was shown to be associated with *A. vaginae*.

### *L. gasseri *and *L. iners*

Overall *L. gasseri *and *L. iners *are abundantly present in most grades. Strikingly, *L. gasseri *is virtually absent in grade III whereas *L. iners *is virtually absent in grades Ia and II.

In our study we found a high concentration of *L. iners *to be clearly associated with low concentrations of *L. gasseri*, which is more prevalent in normal (grade Ia, grade Ib, grade Iab) and mildly disturbed flora (grade II). This was confirmed by Spearman rank testing, which showed that *L. gasseri *and *L. iners *are significantly negatively correlated to each other in grades II and III (*r *= -0.793, p < 0.0001), but also when all samples are considered together (*r *= -0.397, p = 0.001).

It can be noted that even the single grade III sample with a high concentration of *L. gasseri *was also one of the two grade III samples with low concentration of *L. iners*. In addition, we found a positive correlation between *G. vaginalis *and *L. iners*. The distribution frequencies over the different grades of *L. iners *and *G. vaginalis *are quite similar, i.e. present in most grades but predominantly in grade III, but different from *A. vaginae*, i.e. present almost exclusively in grade III, which may explain why *L. iners *does not correlate with *A. vaginae*. This distribution of *L. iners *was also observed in the T-RFLP study of Thies *et al*. [32], with 32 out of 50 BV samples and 11 out of 20 normal microflora samples containing the *L. iners *TRF. The *L. gasseri *group TRF was observed only twice, in women with normal microflora. Unfortunately, these authors did not distinguish between grade II and III.

According to some research groups [35], *L. iners *is the most abundant vaginal *Lactobacillus *species, not only in black African women (64% of 241 healthy premenopausal Nigerian women) but also in white women from Sweden, the US and Canada and is considered as even more typical for normal vaginal microflora than *L. crispatus*. Although *L. iners *was present in high numbers in grades Iab and Ib, we found *L. crispatus *and *L. gasseri *to be more abundant in grade Ia. Ferris *et al*. [17] concluded that *L. iners *is a more transitional species, present in recently cured patients. They reported the predominance of *L. iners *in 5 patients with BV after metronidazole treatment, whereas for the sixth patient, the only one with a complete treatment failure, *L. iners *was present but not predominant. These results were confirmed by Jakobsson and Forsum [36].

Interestingly, it has been observed by the group of Taylor-Robinson that grade II microflora responds poorly to clindamycin treatment whereas most subjects with grade III microflora readily reverted to grade I [37,38] and also that women with clindamycin treated grade III microflora had a better pregnancy outcome than women with clindamycin treated grade II microflora [37]. In agreement, the predominance of clindamycin susceptible *L. iners *in grade III might explain the observations of Taylor-Robinson *et al*. [38].

Finally, in case grade II would be an intermediate stage in between transition from grade I to grade III, one would expect to see an increase in *L. iners *and a decrease in *L. gasseri*, since the former is present in high numbers in grade III and the latter almost absent, but we observe exactly the opposite, i.e. nearly complete absence of *L. iners *and high numbers of *L. gasseri *in grade II.

In order to establish whether this could be related to the differential presence of *L. gasseri *and *L. iners *in these grades, with *L. gasseri *predominant in grade II, we determined the MIC for clindamycin for 10 strains of each. We found that 8 out of 10 *L. iners *strains were inhibited by less than 0.25 μg/ml, whereas 9 out of 10 *L. gasseri *strains had a MIC value of 2 or more μg clindamycin/ml. Although *L. gasseri *cannot be considered definitely as the causative micro-organism responsible for the adverse pregnancy outcome that has been associated with grade II, our data indicate that it can be considered as contributing to the poor response to clindamycin treatment.

To test the hypothesis that *L. gasseri *and *L. iners *are mutually exclusive, e.g. by the production of bacteriocins, we carried out inhibition tests between both species. However, with the method we used we could not observe mutual inhibition between both species.

With the use of molecular techniques it becomes clear that the definition for bacterial vaginosis in which it is claimed that the lactobacilli are replaced by anaerobic bacteria is not quite correct. Our data indicate that rather *L. crispatus*, *L. gasseri *and *L. jensenii *present in a healthy vaginal microflora and grade II microflora are replaced largely by *L. iners *in grade III vaginal microflora. Accordingly, the qualitative culture study by Delaney & Onderdonk [30] found a weak negative correlation between Nugent score and numbers of all lactobacilli, meaning that the number of lactobacillar cells did not decrease with high Nugent score, i.e. disturbed microflora. On the other hand, Zariffard *et al*. [27] and Sha *et al*. [28,29] who quantified lactobacilli in cervicovaginal lavage samples, using a real-time PCR format which picked up only *L. crispatus *and *L. jensenii*, found a decline in the number of lactobacilli in BV. The discrepancy may be explained because we take also *L. iners *and *L. gasseri *into account.

To further resolve these discrepancies we carried out Gram staining of several strains of each *L. gasseri *and *L. iners*. It is of importance to note that *L. iners *has not only long escaped from our attention due to its inability to grow on MRS agar, but also hides as a *Lactobacillus *from Gram stains, because it stains Gram-negative and its cell morphology is rather coccobacillar than bacillar. Since it is the predominant *Lactobacillus *species in BV microflora – even present in high numbers – this has led to the false assumption that the BV microflora is devoid of lactobacilli.

The very long lactobacillar cells observed for some of the *L. gasseri *isolates remind of earlier observations. Pahlson & Larsson [39] reported the occurrence of very long fusiform lactobacilli in 7.4% of the vagina of 981 women studied. Horowitz *et al*. [40] reported the occurrence of 'vaginal lactobacillosis', a pathological condition characterized by the presence of extremely long lactobacilli (up to 60 μm).

Grade I-like was described only recently by Verhelst *et al*. [24] as a grade that, on Gram stain, resembles grade I because of the Gram positive rods, but with the use of molecular methods it was shown that these rods were *Bifidobacterium *spp. instead of *Lactobacillus *spp.

Again this study confirms the separate nature of grade I-like vaginal microflora. A significantly lower concentration of *L. crispatus *(log_10 _MC = 5.5 cells/ml) is present compared to grade I (log_10 _MC = 8.8 cells/ml, p = 0.014) and much lower concentrations of *G. vaginalis *(log_10 _MC = 4.0 cells/ml, p < 0.0001) and *A. vaginae *(log_10 _MC = 1.6 cells/ml, p = 0.003) are present in grade I-like microflora compared to grade III (respectively log_10 _MC = 9.2 cells/ml and log_10 _MC = 9.0 cells/ml).

## Conclusion

We report a strong negative association between *L. iners *and *L. gasseri*, whereas *L. gasseri *is predominant in grade II and *L. iners *in grade III. It has been mentioned by others [5] that a grade II microflora is not necessarily intermediate between grade I and III.

*L. iners *and *L. gasseri *do not inhibit each other by production of bacteriocins, according to the technique we used. In addition we found *L. iners *to be very sensitive for clindamycin (MIC_50 _= 0.125 μg/ml) compared to the higher MIC value for *L. gasseri *(MIC_50 _= 4 μg/ml). Comparison of these results with findings of other research groups is very difficult because *L. iners *has long been overlooked in culture and Gram stain. The unexpected Gram negative pleiomorphic cell shape of *L. iners*, as established in this study, and its inability to grow on MRS agar made the estimation of the occurrence of this species almost impossible until the recent development of molecular techniques.

## Methods

### Strains and culture conditions

The specificity of the primer set for *Lactobacillus iners *was tested using the following strains: *L. iners *(CCUG 28746^T^), *L. brevis *(LB4), *L. delbrueckii *(LB31), *L. crispatus *(ATCC 33199^T^), *L. casei *(ATCC 393^T^), *L. fermentum *(ATCC 23271, ATCC 11739^T^, SAV1, SAV 3, SAV5), *L. gasseri *(ATCC 33323^T^), *L. jensenii *(ATCC 25258^T^), *L. johnsonii *(DSM 20553), *L. plantarum *(ATCC 8014, ATCC 10012, ATCC 14917^T^), *L. reuteri *(ATCC 23272^T^, RC-14), *L. rhamnosus *(ATCC 7469^T^, GR-1, GG) and *L. salivarius *(SAV6).

The specificity of the primer set for *Lactobacillus jensenii *was tested using the following strains:*L. acidophilus *(LMG 11430), *L. animalis *(LMG 9843^T^), *L. bifermentans *(LMG 11431), *L. brevis *(LMG 7761), *L. casei *(FB86-16), *L. coleohominis *(PB2003/024-T1-1a), *L. crispatus *(LMG 9479^T^), *L. curvatus *(0606 93811c), *L. delbrueckii *(LMG 6412^T^)*, L. fermentum *(LMG 6902^T^), *L. gasseri *(LMG 9203^T^), *L. helveticus *(LBP152), *L. iners *(BVS11, PB2003/044-T1-1, PB2003/53-T1-1), *L. intestinalis *(LMG 14196^T^), *L. jensenii *(PB2003/204-T1-1, FB65-6, FB122-BP-1, FB146-BA-5, FB154-CAN-2), *L. johnsonii *(LBP151), *L. plantarum *(LMG 9212), *L. reuteri *(LMG 9213T), *L. rhamnosus *(LMG 8153, LMG18243), *L. salivarius *(LMG 9477^T^) and *L. vaginalis *(LMG 12891^T^).

The specificity of the primer set for *Gardnerella vaginalis *was tested using the following strains: *Bifidobacterium infantis *(PB2003/126-T1-3), *B. breve *(PB2003/108-T1-1) and *B. bifidum *(PB2003/99-T1-2).

The specificity of the primer set for *Atopobium vaginae *was tested using the following strains: *A. minutum *(CCUG 31167), *A. parvulum *(CCUG 32760), *A. rimae *(CCUG 31168) and *A. vaginae *(CCUG 38953^T^, CCUG 42099, CCUG 44116, CCUG 44125, PB2003/189-T1-4). All strains were cultured on Trypticase soy agar with 5% sheep blood (Becton Dickinson, Erembodegem, Belgium) in anaerobic conditions (Gaspak, Becton Dickinson) for 72 hours at 37°C.

### Samples

A total of 142 swabs (Copan, Brescia, Italy) were collected by sampling 60 women of childbearing age (32 pregnant women, 28 non-pregnant women). Sampling was carried out by insertion of two sterile cotton swabs into the vaginal vault, after placement of a non-lubricated speculum. The swabs were rotated against the vaginal wall at the mid-portion of the vault and were carefully removed to prevent contamination with microflora of the vulva and introitus. The first swab was used to prepare a smear on a glass slide for the purpose of grading as described by Verhelst *et al*. [14]. The second swab was returned to a sterile tube for the purpose of DNA extraction (dry swab). Both swabs were sent to the microbiology laboratory and were processed within 4 hours.

The study protocol was approved by the ethical committee of the Ghent University Hospital and individual written informed consent was obtained.

### DNA extraction of bacteria and samples

DNA was extracted from cultured bacteria by simple alkaline lysis. One colony was suspended in 20 μl of lysis buffer (0.25% SDS, 0.05 N NaCl and 95 ml sterile distilled water) and heated for 15 minutes at 95°C. After briefly centrifugating, 180 μl of HPCL-water was added and this was centrifuged again for 5 minutes at 16300 g. Concentrations of DNA-extracts ranged between 2.1 and 87.5 ng/μl.

For dry vaginal swabs, the QIAamp DNA mini kit (Qiagen, Hilden, Germany) was used according to the manufacturer's recommendations, with minor modifications. The dry swab specimen from each patient was swirled for 15 s in 400 μl of lysis buffer (20 mM Tris-HCl, pH 8.0; 2 mM EDTA; 1.2% Triton). Fifty units of mutanolysin (25 U/μl) (Sigma, Bornem, Belgium) were added and the samples were incubated for 30 min at 37°C. After the addition of 20 μl Proteinase K (20 mg/ml) and 200 μl AL buffer (Qiagen), samples were incubated for 30 min at 56°C. Next, 200 μl of ethanol was added and DNA was purified by adding the lysate to the Qiagen columns as described by the manufacturer. Finally, the total bacterial DNA was eluted with 100 μl of AE buffer (Qiagen). DNA extracts were aliquoted and stored at -20°C.

### Primers

PCR primer sets targeting *L. iners*, *L. jensenii *and *A. vaginae *were designed by aligning the sequences of the 16S rRNA gene of different strains of both species by CLUSTALW [41]. Specificity of the primer sets (Eurogentec, Seraing, Belgium) was analyzed by using the BLAST algorithm [42]. Primer sequences for *L. jensenii *and *L. iners *alignment to the most common vaginal lactobacilli are shown in Table 1 and Table 2. Primer sequences for *A. vaginae *and alignment to other members of the *Atopobium *group are shown in Table 3. Position of the primers on the 16S rRNA-gene is shown on Figure 1. Optimal annealing temperature of each primer set was established by gradient PCR and analysis on an agarose gel. In addition, already described primers for *L. crispatus *and *L. gasseri *[26] and *G. vaginalis *[27] were used. Primer sequences and cycling conditions are summarized in Table 4. Primer concentrations were 100 nM each except for the assays for *G. vaginalis *and *L. iners *for which the primer concentrations were 200 nM.

### Construction of the standard curves for real-time PCR

To construct standard curves for the real-time PCR's, *A. vag*inae (CCUG 38953^T^), *L. crispatus *(PB2003/125-T1-1), *L. gasseri *(FB102-1) and *L. jensenii *(PB2003/204-T1-1) were cultured on TSA + 5% sheep blood (Becton Dickinson) and *G. vaginalis *(LMG 7832^T^) and *L. iners *(BVS11) were cultured on CNA agar (Becton Dickinson) + 5% human blood. A suspension was made in MRS Broth (Oxoid, Drongen, Belgium) and DNA was extracted. The DNA concentration of this stock was determined ten times by using the Nanodrop ND-1000 (Nanodrop Technologies, Wilmington, USA) and the mean value was used for further calculations. For each strain a tenfold dilution series was prepared by dilution of the DNA stock in HPLC grade water. Dilutions were aliquoted and stored frozen at -20°C.

### Real-time PCR

The qPCR Core Kit for SYBR Green I (Eurogentec) was applied and analysis was performed on the ABI 7300 real-time PCR system (Applied Biosystems, Foster City, CA).

Reactions were done in PCR mixtures containing 2.5 μl of DNA extract, 2.5 μl of 10 × Reaction Buffer, 3.5 mM MgCl_2_, 0.2 mM dNTP mixture, 0.625 U HotGoldStar *Taq *polymerase, 0.75 μl SYBR^® ^Green I, diluted 10-fold in DMSO and the appropriate primer concentration, adjusted with HPLC grade water to 25 μl. Primer concentrations are summarized in Table 4. Each run included a standard curve and each sample was run in triplicate. In case the result was not in the range of the standard curve, the samples were diluted tenfold and analyzed in triplicate again. The median log_10 _cells/ml were expressed as per 1 ml elution buffer.

### Agar dilution method

Clindamycin (Sigma, Bornem, Belgium) minimal inhibitory concentrations (MIC) were determined for 10 strains of *L. gasseri (*FB02-02, FB13-01, FB29-6, FB34-01AN, FB86-6, FB89-3, FB102-1b, FB103-2, PB88-T2-1 and PB327-1) and 10 strains of *L. iners *(BVS11, FB04-04, FB05-01, FB06-01, FB07-01, FB17-07, FB30-01AN, FB77-03, FB94-05b and FB123-CNA-4) using the agar dilution method on Trypticase Soy Agar (Becton Dickinson) + 5% human blood and incubation anaerobically during 72 hours at 37°C in the Anaerobic Workstation BugBoxPlus (LED Techno, Heusden-Zolder, Belgium). Concentrations of 8, 4, 2, 1, 0.5, 0.250, 0.125 and 0.0625 μg/ml, were tested for each strain in triplicate.

### Mutual inhibition test

The same strains used for MIC-determination were grown in Trypticase Soy Broth (Becton Dickinson) + 5% human blood for 72 hours at 37°C in the anaerobic workstation BugBoxPlus (LED Techno, Heusden-Zolder, Belgium). Supernatant of the liquid cultures and of the negative control were sterilized trough a 0.22 μm filter (Spin-X Centrifuge Tube Filter, Costar, Corning, NY14831). Plates were inoculated with a swab to obtain confluent growth. Volumes of 20 μl of the filter sterilized supernatants were dropped onto the inoculated plates, followed by anaerobic incubation at 37°C for 72 hours, whereafter inhibition zones were recorded.

### Data analysis

For the calculation of number of cells in each dilution, genome size and % G+C of the bacterial species was taken into account. The genome size of *G. vaginalis *(ATCC 14019, ATCC 14018, GVP 007 and GVP 004) has been approximated to 1.7 Mb [43] and % G+C was 43 [44]. For *L. gasseri *data described for strain ATCC 33323 (genome size: 1.9 Mb, 35.3% G+C) [45] were used. Also for *A. vaginae *strain CCUG 38953^T ^(44% G+C) [46], *L. crispatus *strain ATCC 33820^T ^(35% G+C) [47], *L. iners *strain CCUG 28746^T ^(34.4% G+C) [20] and *L. jensenii *strain ATCC 25258^T ^(36.1% G+C) [48] a genome size of 1.9 Mb was assumed.

### Statistical analysis

Data were analyzed under the non-parametric assumption, taking into consideration the log_10 _[count] distributions of species under study did not approximate the normal distribution. For any given category (grades I-IV) the distribution of concentrations (log_10 _cells/ml) of each species was expressed as the median count and the accompanying interquartile range (IQR). Between group comparisons of distributions were performed with the Mann-Whitney U-test for two groups and with the Kruskal Wallis test for multiple groups. Correlations between the different species were determined by the Spearman (rank) test and reported as Spearman's rho value (r). All analyses were performed using SPSS v15 software (Chicago, Illinois).

## Authors' contributions

EDB, RV and MV participated in the development of the study design, the analysis of the study samples, the collection, analysis and interpretation of the data, and in the writing of the report. HV and MT participated in the development of the study design, the collection of the study samples, the collection, analysis and interpretation of the data, and in the writing of the report. MAQ, JB and JRT developed the *L. iners *primers, participated in analysis and interpretation of the data, and in the writing of the report. All authors read and approved the final manuscript.

## References

[B1] Morris M, Nicoll A, Simms I, Wilson J, Catchpole M (2001). Bacterial vaginosis: a public health review. BJOG.

[B2] Haggerty CL, Hillier SL, Bass DC, Ness RB (2004). Bacterial vaginosis and anaerobic bacteria are associated with endometritis. Clin Infect Dis.

[B3] Ness RB, Kip KE, Hillier SL, Soper DE, Stamm CA, Sweet RL, Rice P, Richter HE (2005). A cluster analysis of bacterial vaginosis-associated microflora and pelvic inflammatory disease. Am J Epidemiol.

[B4] Watts DH, Krohn MA, Hillier SL, Eschenbach DA (1990). Bacterial vaginosis as a risk factor for post-cesarean endometritis. Obstet Gynecol.

[B5] Hay PE, Lamont RF, Taylor-Robinson D, Morgan DJ, Ison C, Pearson J (1994). Abnormal bacterial colonisation of the genital tract and subsequent preterm delivery and late miscarriage. BMJ.

[B6] Hillier SL, Nugent RP, Eschenbach DA, Krohn MA, Gibbs RS, Martin DH, Cotch MF, Edelman R, Pastorek JG, Rao AV (1995). Association between bacterial vaginosis and preterm delivery of a low-birth-weight infant. The Vaginal Infections and Prematurity Study Group. N Engl J Med.

[B7] Leitich H, Bodner-Adler B, Brunbauer M, Kaider A, Egarter C, Husslein P (2003). Bacterial vaginosis as a risk factor for preterm delivery: a meta-analysis. Am J Obstet Gynecol.

[B8] Pretorius C, Jagatt A, Lamont RF (2007). The relationship between periodontal disease, bacterial vaginosis, and preterm birth. J Perinat Med.

[B9] Martin HL, Richardson BA, Nyange PM, Lavreys L, Hillier SL, Chohan B, Mandaliya K, Ndinya-Achola JO, Bwayo J, Kreiss J (1999). Vaginal lactobacilli, microbial flora, and risk of human immunodeficiency virus type 1 and sexually transmitted disease acquisition. J Infect Dis.

[B10] Sewankambo N, Gray RH, Wawer MJ, Paxton L, McNaim D, Wabwire-Mangen F, Serwadda D, Li C, Kiwanuka N, Hillier SL, Rabe L, Gaydos CA, Quinn TC, Konde-Lule J (1997). HIV-1 infection associated with abnormal vaginal flora morphology and bacterial vaginosis. Lancet.

[B11] Hawes SE, Hillier SL, Benedetti J, Stevens CE, Koutsky LA, Wolner-Hanssen P, Holmes KK (1996). Hydrogen peroxide-producing lactobacilli and acquisition of vaginal infections. J Infect Dis.

[B12] Swidsinski A, Mendling W, Loening-Baucke V, Ladhoff A, Swidsinski S, Hale LP, Lochs H (2005). Adherent biofilms in bacterial vaginosis. Obstet Gynecol.

[B13] Ferris MJ, Masztal A, Aldridge KE, Fortenberry JD, Fidel PL, Martin DH (2004). Association of *Atopobium vaginae*, a recently described metronidazole resistant anaerobe, with bacterial vaginosis. BMC Infect Dis.

[B14] Verhelst R, Verstraelen H, Claeys G, Verschraegen G, Delanghe J, Van Simaey L, De Ganck C, Temmerman M, Vaneechoutte M (2004). Cloning of 16S rRNA genes amplified from normal and disturbed vaginal microflora suggests a strong association between *Atopobium vaginae*, *Gardnerella vaginalis* and bacterial vaginosis. BMC Microbiol.

[B15] Burton JP, Devillard E, Cadieux PA, Hammond JA, Reid G (2004). Detection of *Atopobium vaginae* in postmenopausal women by cultivation-independent methods warrants further investigation. J Clin Microbiol.

[B16] Bradshaw CS, Tabrizi SN, Fairley CK, Morton AN, Rudland E, Garland SM (2006). The association of *Atopobium vaginae* and *Gardnerella vaginalis* with bacterial vaginosis and recurrence after oral metronidazole therapy. J Infect Dis.

[B17] Ferris MJ, Norori J, Zozaya-Hinchliffe M, Martin DH (2007). Cultivation-independent analysis of changes in bacterial vaginosis flora following metronidazole treatment. J Clin Microbiol.

[B18] Hay PE (1998). Recurrent bacterial vaginosis. Dermatol Clin.

[B19] Antonio MA, Hawes SE, Hillier SL (1999). The identification of vaginal *Lactobacillus* species and the demographic and microbiologic characteristics of women colonized by these species. J Infect Dis.

[B20] Falsen E, Pascual C, Sjoden B, Ohlen M, Collins MD (1999). Phenotypic and phylogenetic characterization of a novel *Lactobacillus* species from human sources: description of *Lactobacillus iners* sp. nov. Int J Syst Bacteriol.

[B21] Spiegel CA, Amsel R, Holmes KK (1983). Diagnosis of bacterial vaginosis by direct gram stain of vaginal fluid. J Clin Microbiol.

[B22] Nugent RP, Krohn MA, Hillier SL (1991). Reliability of diagnosing bacterial vaginosis is improved by a standardized method of gram stain interpretation. J Clin Microbiol.

[B23] Ison CA, Hay PE (2002). Validation of a simplified grading of Gram stained vaginal smears for use in genitourinary medicine clinics. Sex Transm Infect.

[B24] Verhelst R, Verstraelen H, Claeys G, Verschraegen G, Van Simaey L, De Ganck C, De Backer E, Temmerman M, Vaneechoutte M (2005). Comparison between Gram stain and culture for the characterization of vaginal microflora: definition of a distinct grade that resembles grade I microflora and revised categorization of grade I microflora. BMC Microbiol.

[B25] Verstraelen H, Verhelst R, Roelens K, Claeys G, Weyers S, De Backer E, Vaneechoutte M, Temmerman M (2007). Modified classification of Gram-stained vaginal smears to predict spontaneous preterm birth: a prospective cohort study. Am J Obstet Gynecol.

[B26] Byun R, Nadkarni MA, Chhour KL, Martin FE, Jacques NA, Hunter N (2004). Quantitative analysis of diverse *Lactobacillus* species present in advanced dental caries. J Clin Microbiol.

[B27] Zariffard MR, Saifuddin M, Sha BE, Spear GT (2002). Detection of bacterial vaginosis-related organisms by real-time PCR for *Lactobacilli*, *Gardnerella vaginalis* and *Mycoplasma hominis*. FEMS Immunol Med Microbiol.

[B28] Sha BE, Chen HY, Wang QJ, Zariffard MR, Cohen MH, Spear GT (2005). Utility of Amsel criteria, Nugent score, and quantitative PCR for *Gardnerella vaginalis*, *Mycoplasma hominis*, and *Lactobacillus* spp. for diagnosis of bacterial vaginosis in human immunodeficiency virus-infected women. J Clin Microbiol.

[B29] Sha BE, Zariffard MR, Wang QJ, Chen HY, Bremer J, Cohen MH, Spear GT (2005). Female genital-tract HIV load correlates inversely with *Lactobacillus* species but positively with bacterial vaginosis and *Mycoplasma hominis*. J Infect Dis.

[B30] Delaney ML, Onderdonk AB (2001). Nugent score related to vaginal culture in pregnant women. Obstet Gynecol.

[B31] Ferris MJ, Masztal A, Martin DH (2004). Use of species-directed 16S rRNA gene PCR primers for detection of *Atopobium vaginae* in patients with bacterial vaginosis. J Clin Microbiol.

[B32] Thies FL, Konig W, Konig B (2007). Rapid characterization of the normal and disturbed vaginal microbiota by application of 16S rRNA gene terminal RFLP fingerprinting. J Med Microbiol.

[B33] Burton JP, Chilcott CN, Al-Qumber M, Brooks HJ, Wilson D, Tagg JR, Devenish C (2005). A preliminary survey of *Atopobium vaginae* in women attending the Dunedin gynaecology out-patients clinic: is the contribution of the hard-to-culture microbiota overlooked in gynaecological disorders?. Aust N Z J Obstet Gynaecol.

[B34] De Backer E, Verhelst R, Verstraelen H, Claeys G, Verschraegen G, Temmerman M, Vaneechoutte M (2006). Antibiotic susceptibility of *Atopobium vaginae*. BMC Infect Dis.

[B35] Anukam KC, Osazuwa EO, Ahonkhai I, Reid G (2006). *Lactobacillus* vaginal microbiota of women attending a reproductive health care service in Benin city, Nigeria. Sex Transm Dis.

[B36] Jakobsson T, Forsum U (2007). *Lactobacillus iners*; a marker of changes in the vaginal flora?. J Clin Microbiol.

[B37] Rosenstein IJ, Morgan DJ, Lamont RF, Sheehan M, Dore CJ, Hay PE, Taylor-Robinson D (2000). Effect of intravaginal clindamycin cream on pregnancy outcome and on abnormal vaginal microbial flora of pregnant women. Infect Dis Obstet Gynecol.

[B38] Taylor-Robinson D, Morgan DJ, Sheehan M, Rosenstein IJ, Lamont RF (2003). Relation between Gram-stain and clinical criteria for diagnosing bacterial vaginosis with special reference to Gram grade II evaluation. Int J STD AIDS.

[B39] Pahlson C, Larsson PG (1991). The ecologically wrong vaginal lactobacilli. Med Hypotheses.

[B40] Horowitz BJ, Mardh PA, Nagy E, Rank EL (1994). Vaginal lactobacillosis. Am J Obstet Gynecol.

[B41] Thompson JD, Higgins DG, Gibson TJ (1994). CLUSTAL W: improving the sensitivity of progressive multiple sequence alignment through sequence weighting, position-specific gap penalties and weight matrix choice. Nucleic Acids Res.

[B42] Altschul SF, Gish W, Miller W, Myers EW, Lipman DJ (1990). Basic local alignment search tool. J Mol Biol.

[B43] Lim D, Trivedi H, Nath K (1994). Determination of *Gardnerella vaginalis* genome size by pulsed-field gel electrophoresis. DNA Res.

[B44] Eschenbach DA (1993). Bacterial vaginosis and anaerobes in obstetric-gynecologic infection. Clin Infect Dis.

[B45] Makarova K, Slesarev A, Wolf Y, Sorokin A, Mirkin B, Koonin E, Pavlov A, Pavlova N, Karamychev V, Polouchine N, Shakhova V, Grigoriev I, Lou Y, Rohksar D, Lucas S, Huang K, Goodstein DM, Hawkins T, Plengvidhya V, Welker D, Hughes J, Goh Y, Benson A, Baldwin K, Lee JH (2006). Comparative genomics of the lactic acid bacteria. Proc Natl Acad Sci U S A.

[B46] Rodriguez Jovita M, Collins MD, Sjoden B, Falsen E (1999). Characterization of a novel *Atopobium* isolate from the human vagina: description of *Atopobium vaginae* sp. nov. Int J Syst Bacteriol.

[B47] Cato EP, C MWE, Johnson JL (1983). Synonymy of strains of "*Lactobacillus acidophilus*" group A2 (Johnson et al. 1980) with the type strain of *Lactobacillus crispatus* (Brygoo and Aladame 1953) Moore and Holdeman 1970.. Int J Syst Bacteriol.

[B48] Gasser F, Mandel M, Rogosa M (1970). *Lactobacillus jensenii* sp.nov., a new representative of the subgenus Thermobacterium. J Gen Microbiol.

